# Dynamics and detection of the Newton-Wigner time delays at interfaces using a swivelling method

**DOI:** 10.1038/s41598-017-09502-9

**Published:** 2017-08-22

**Authors:** Albert Le Floch, Olivier Emile, Guy Ropars, Govind P. Agrawal

**Affiliations:** 1 Laboratoire de Physique des Lasers, UFR SPM, Université de Rennes 1, 35042 Rennes, France; 2 Université Bretagne Loire, 35044 Rennes, France; 3 Laboratoire d’Electronique Quantique et Chiralités, 20 square Marcel Bouget, 35700 Rennes, France; 4 The Institute of Optics, University of Rochester, Rochester, New York, 14627 USA

## Abstract

Evanescent waves are ubiquitous at interfaces with optical, seismic or acoustic waves, and also with electron, neutron or atom beams. Newton was the first to suspect that both small time delays and spatial shifts exist during total internal reflection. However, these effects are so tiny that the spatial shifts were only observed in 1947 in optics, whereas the time delay values predicted by the Wigner model in the 10^−14^ s range in optics had to await femtosecond lasers to be detected with difficulty. The spatial shifts have been isolated in many areas but the time delays, though fundamental, generally remain out of reach, particularly with particles. In textbooks usually both quantities are supposed to be simply linked. Here we report, using swivelling detectors, that the spatial and temporal measurements are intimately intermingled, especially in the so-called cyclical regime. Indeed, while the spatial shift does not depend on the type of detection, the measured time delay can be positive, negative or zero, but controllable. We also discuss how such intricate measurements of spatial and temporal effects allow crucial time penalties to be eliminated in guided soliton propagation, and should be used to unambiguously identify the Newton-Wigner time delays for particles.

## Introduction

**Figure 1:**
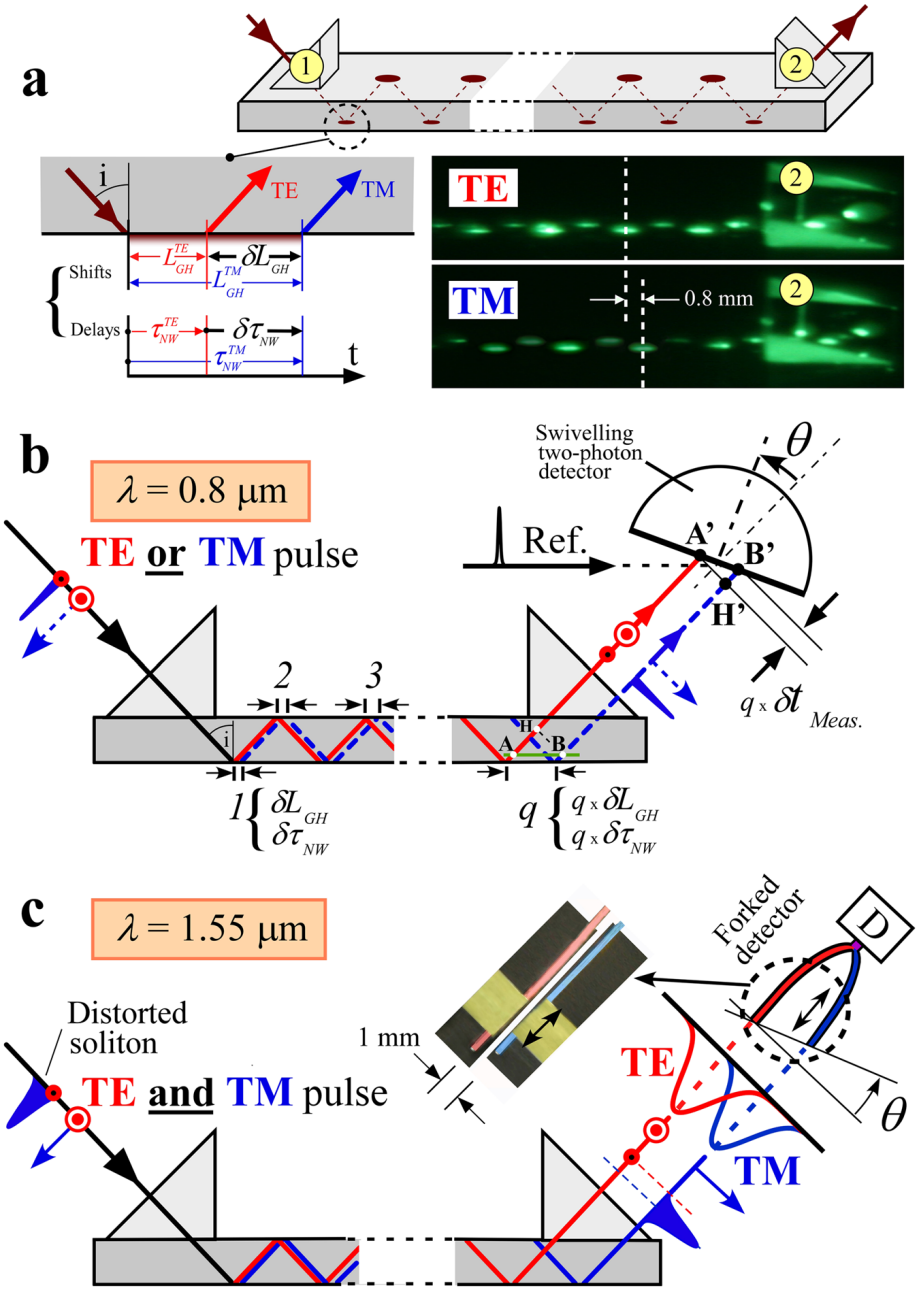
Goos-Hänchen shifts and Newton-Wigner time delays in different regimes. (**a**) A Ti:Sa laser beam at a wavelength of 808 nm is coupled into a waveguide via the prism 1 and undergoes up to 161 total internal reflections before being outcoupled by the prism 2. The laser could operate either in CW or pulsed regime. The total Goos-Hänchen shifts between TE and TM beams reaches 0.8 mm. It is directly observed through an infrared viewer giving the green colour. (**b**) In the femto-second regime, the Newton-Wigner delay *qδτ*_*Meas*._ is measured by a correlation with a fixed reference beam on a two-photon detector, whose surface of detection could be rotated by an angle *θ*. The pulse polarizations are successively TE or TM. Note that for *θ* = 0°, the TE and TM pulses arrive at the same time due to an automatic compensation in the second prism. **(c)** In the soliton regime, when a randomly polarized soliton at a wavelength of 1550 nm is launched into a telecommunication system including a waveguide, a forked fibre detector can take advantage of the intermingled spatial and temporal shifts to compensate for the distortion.

Although suggesting a mechanical corpuscular model of light, Newton was the first to observe and use evanescent waves at total reflection^1^. Moreover he suggested spatial shifts and time delays for light impinging on an interface. Among the many definitions of time delays^2^, considering the energy derivative of the scattered phase shift δ during an elastic collision, Wigner introduced a time delay value *τ* linked to the principle of causality^3^, in the form ${\rm{\tau }}=\hslash \,\frac{\partial {\rm{\delta }}}{\partial {\rm{E}}}$. This formulation has been extended to space-time intervals^4^ and applied to the total reflection case^5^, correlating the expected Newton-Wigner time delay, i.e., the time spent in the second medium, to the Goos-Hänchen spatial shift^6^. This spatial shift δ*L*_*GH*_ (see Fig. 1a) has been extensively investigated, both theoretically and experimentally, at interfaces involving not only all kinds of optical media such as glass^7^, metals^8^, superconductors^9^, nematics^10^, graphene^11^, magnetic materials^12^ but also using newly discovered metamaterials with properties not available in nature^13–16^. The Goos-Hänchen shift has also been observed in acoustics^17, 18^, and neutronics^19^ and is expected in seismology^20^ and in quantum reflection^21^. However, from the experimental point of view, while the spatial shift requires only a continuous wave (CW) set-up to be observed, measuring the Newton-Wigner time delay (in the range of 10^−14^ s in optics) necessitates short pulses, a reference clock, and an appropriate finish line for the arrival of two pulses, when two spatially separated trajectories are used. The measurement of such delays requires detecting the difference between the time arrivals of two pulses propagating along parallel pathways. As for runners in different lanes in a stadium, one has to clearly identify the start and finish lines. Fundamentally, spatial shifts and time delays are expected for transverse, longitudinal and de Broglie matter waves, but the two measured quantities are experimentally structurally intermingled (Fig. 1).

In recent years the Newton-Wigner time delays at interfaces have been observed with difficulty in optics for gratings and dielectrics using complex techniques^22–24^. Indeed, for a single reflection on an interface, only differential methods using synchronous detections allow one to reach the expected small Newton-Wigner delays of about 20 fs between broadened 1000 fs TE and TM correlation signals^24^. In fact, in any experiment, one has to compare the pulse arrivals for two beams with spatially separated pathways, with their own possible extra delays. On the one hand, in contrast to the measurement of spatial shifts that remain unchanged in a continuous wave or a pulsed regime, the measurement of time delays requires a pulse regime with short optical pulses and is expected to exhibit experimentally paradoxical dynamics, according to the chosen finish line. For this reason, spatial shifts and the measured delays cannot be simply linked as described in textbooks^25, 26^. Nonetheless, on the other hand, combining spatial shifts with time delays could provide us with the possibility to compensate for unwanted penalties occurring in fast phenomena such as soliton propagation. Today, temporal solitons are the subject of intense research^10, 27, 28^ and find applications in many areas including communications and frequency combs with their numerous uses beyond the laboratory environment. In systems where temporal solitons interact with interfaces, combining the spatial shifts with time delays opens new possibilities to control soliton perturbations. Moreover, such a combination could help us in unambiguously identifying time delays in the particle domain.

Beyond the difficulties to access the delays, several experiments^22–24^ have used counterintuitive schemes, where the detection line is oriented parallel to the interface.

Modern nanophotonic techniques have been proposed to reach Newton-Wigner delays by simply orienting the detection plane perpendicular to the beam propagation^29^. Unfortunately, in this case there is a mathematical compensation of the Newton-Wigner delays^22^ due to the inherent extra optical length in the glass^22, 24^. Indeed, around the critical angle at total reflection in glass (see Fig. 1b), the time delay *qδτ*_*NW*_ of the TM pulse along the interface (along AB) is exactly compensated by the extra TE pulse propagation time from A to H in the glass:

1 $$q\delta {\tau }_{NW}=(n/c)\cdot q\delta {L}_{{GH}}\cdot sin\,i,$$

where *n* is the refractive index of the waveguide, *i* is the angle of incidence, and *sin i* = *1/n*.

The observed residual time delay in ref. 29 is probably due to an unavoidable pulse distortion in the attenuated total reflection system, which is not related to Goos-Hänchen shifts and Newton-Wigner delays studied here. The preceding discussion shows the difficulty to reach the Newton-Wigner time delay itself. This is particularly true, as even orienting the detection plane parallel to the interface^22–24^ is not universal. In fact, as shown below, when the Goos-Hänchen shift vanishes like in the so-called cyclical regime, surprisingly, orienting the detection plane perpendicular to the beam axis works correctly to measure the delay. Hence, to reach the fundamental Newton-Wigner time delay in a given experiment, we have to use a swivelling method. An experimental set-up has to fulfil two conditions: i) be based on a direct method, avoiding complex modulation techniques, so as to clearly identify the different pulse arrival times, ii) meet the Rayleigh-like criterion which requires that the time interval between the two correlation signals is at least equal to the half-width at half-maximum of the correlation signal itself.

## Materials and Methods

In optics, the two preceding conditions can only be satisfied by amplifying the shifts and delays of femtosecond laser pulses in a planar guiding slab of refractive index *n* as shown in Fig. 1a. In this case the geometrical properties of the Gaussian beam can be perfectly preserved even after *q* = 161 successive reflections by choosing the angle of incidence just above the critical angle of total internal reflection. The resulting large 0.8 mm spatial shifts *qδL*_*GH*_ between the TM and TE polarized beams can then be seen even with the naked eye (see photo of Fig. 1a taken near the output prism 2). Unfortunately, directly measuring the corresponding time delay *qδτ*_*NW*_ between the TM and TE pulses at the interface, would require placing a detector parallel to the interface along the line AB (see Fig. 1b) just after the last reflection inside the denser medium, i.e. in the glass, which is impossible. A second prism is necessary to extract the pulses after *q* reflections, adding extra delays in the measurements. Alternative experimental configurations can be imagined along the surface, in the air medium. For example, the detector could be butt coupled to the waveguide, or ultrafast near-field microscopy could be used, and the angle of incidence varied. Both cases require a reference signal, and the adjustments of the angle of incidence are tricky around the critical angle and cannot give access to the same dynamics. In our case, the two required conditions are fulfilled using a Ti:Sapphire laser delivering 150 fs pulses (about 8 nm Fourier-limited bandwidth). Indeed for an expected delay of about 3000 fs along the surface after 161 successive reflections, a direct correlation with a reference beam, taking into account of the geometry of Fig. 1b, will give separated signals with a half-width at half-maximum of about 300 fs (larger than any pulse broadening due to the chromatic dispersion in the waveguide), satisfying the Rayleigh-like criterion.

The planar waveguide consists of a 30 cm long, 5 cm wide and 0.15 cm thick silica plate with a surface flatness of *λ*/2. Two 0.5 cm right-angle prisms used to couple the pulses in and out are also made of fused silica. The refractive index of the plate and of the prisms equals 1.453. An index matching liquid at the interfaces between the plate and the prisms and a careful adjustment of the position of the prisms, enable us to keep the optical quality of the 400 μm laser beam waist, even after 161 reflections (see photograph of Fig. 1). We use two Newport M-URM80MS microstage rotations which have an angular resolution of 0.001°. The first one enables the angle of incidence to be set at *i* = 43.53°. The second one is used to rotate the swivelling detectors. For the time delay, we used a Hamamatsu G1116 GaAsP photodiode which has a band gap around 680 nm. This photodiode exhibits a nonlinear two-photon absorption at 800 nm. In order to observe the dynamics of the delays, the reference beam is kept constant while the swivelling detector is rotated (see Fig. 1b). For detecting the spatial shifts, the two-photon photodiode is replaced by a Hamamatsu S11071-1004 CCD image sensor with 1024 elements spaced by 25 μm.

**Figure 2:**
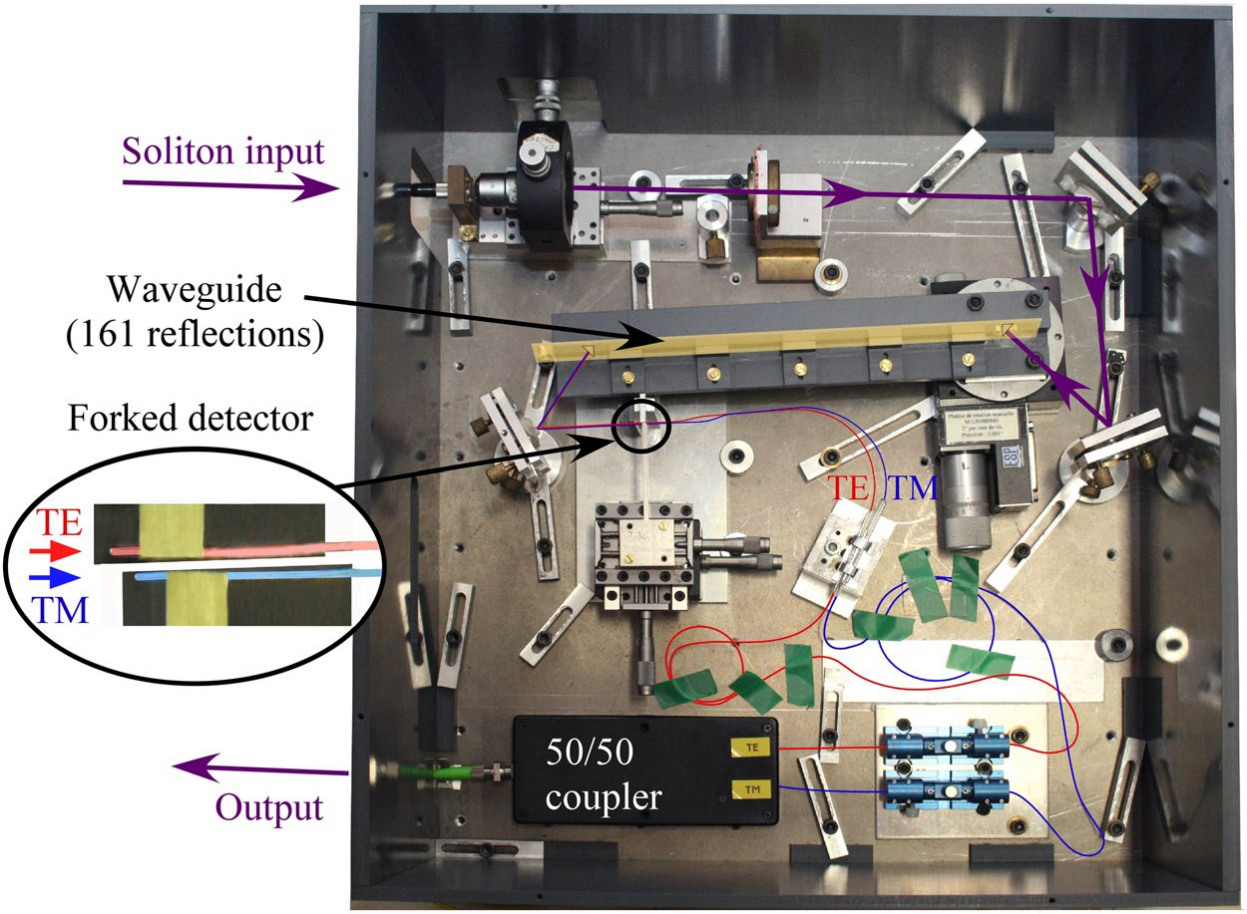
Experimental setup for the soliton experiment. Photograph of the telecommunication system for *λ*  = 1550 nm including the plane waveguide. The swivelling detection is performed here by the forked detector. The red and the blue fibres carry the TE and the TM beams respectively.

To compensate for a soliton distortion we use another swivelling method with the fibre optics shown in Fig. 2. In the forked detector (see Fig. 1c), the 1 mm distance between the two fibres is adjusted so as to correspond to the spatial shift between the TE and TM beams through the waveguide. The TE and TM pulses then propagate within two separate fibres before reaching the detector. The distance between the output of the waveguide and the fibre input faces can be adjusted independently for the two fibres to compensate for the difference in the arrival times of the TE and TM pulses, using a microstage translator. The two input faces of the fibres define a finish line, as when we dealt with the femtosecond pulses. A laser oscillating at 1550 nm generates 2.5 ps soliton pulses with a repetition rate of 10 GHz. These pulses are electrically time multiplexed to obtain a 40 GHz repetition rate. The telecommunication signal is encoded via an electro-optic modulator to get a return-to-zero modulation format at 40 Gbit/s. This signal is finally optically multiplexed four times to obtain a 160 Gbit/s telecommunication signal. After the waveguide is inserted in the system (Fig. 2), the two TE and TM components of the soliton are sent into the forked detector. Its output is connected to a standard 50/50 coupler, before entering an EXFO PSO-102 optical sampling oscilloscope.

## Results and Discussion

### The comparative dynamics of the Goos-Hänchen shifts and the Newton-Wigner delays

**Figure 3:**
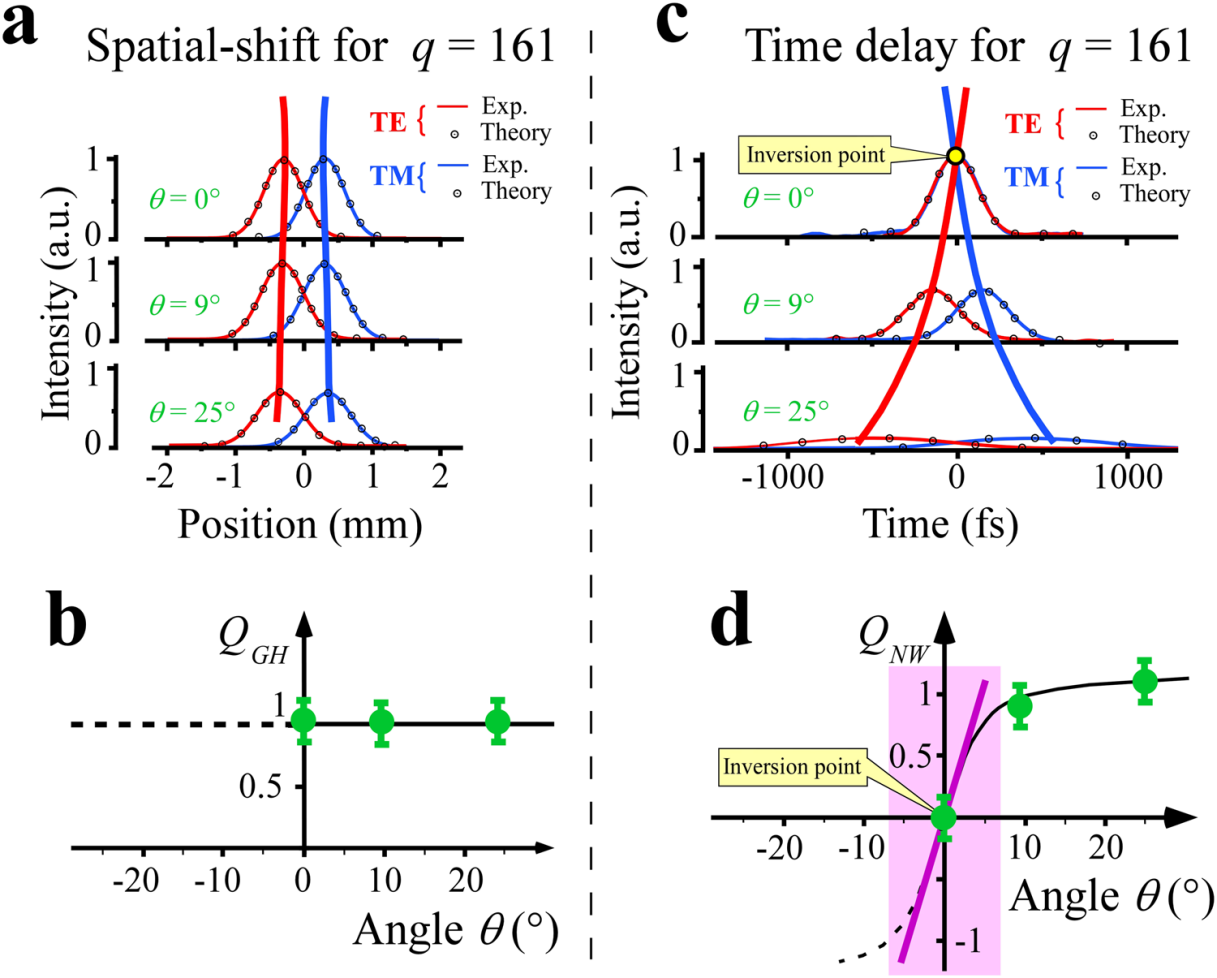
Respective dynamics of the measured spatial shifts and delay times. **(a)** Experimental and theoretical dynamics of the spatial shifts for the TE and TM polarizations for different orientations of the finish line in the plane of incidence of the waveguide. **(b)** Plot of the figure of merit *Q* for the different measurements showing the invariance of the *Q* factor versus the orientation of the detection line. (**c**) Corresponding responses for the delay times. The measured value for *θ* = 9° leads to a Newton-Wigner delay value along the interface of 2595 ± 30 fs. Note here, the existence of an inversion point where the sign of the measured delay times changes. **(d)** The corresponding factor of merit *Q* shows large variations around the inversion point schematized by the purple line.

To investigate the link between the spatial shifts and the measured time delays, let us compare their respective dynamics in the guided setup of Fig. 1b, using the swivelling detectors. The experimental results are shown in Fig. 3a for three orientations of the detector demonstrating the stability and the robustness of the measured values of the spatial shifts. When the detection angle *θ* is increased, the measured Goos-Hänchen signals are only slightly spread by (*cosθ*)^−1^. For *θ* = 25° orientation, the shifts of the Gaussian beams are increased by 10%. However, if we introduce a factor of merit, *Q*_*GH*_ = *q δL*_*GH*_/*Δω* where *Δω* corresponds to the measured width of the Gaussian beam at half-maximum, *Q*_*GH*_ remains constant as shown in Fig. 3b. The measurements of the spatial shifts are stable and unchanged in both the CW and pulse regimes.

Measuring the corresponding time delays is more tricky. As shown in Fig. 1b, we have to use a two-photon detector so as to detect the correlation of a fixed reference pulse with the TE and the TM pulses successively. However, although the TM pulse spends more time outside the glass than the TE pulse, at total reflection, no delay is observed for *θ* = 0° (Fig. 3c). Indeed, after q reflections, the TE pulse is reflected before the TM pulse (see Fig. 1b). As already stated in Eq. 1, the Newton-Wigner delay for the TM pulse is equal to the propagation delay of the TE pulse from A to H, i.e.

2 $${\rm{AH}}\cdot (n/c)=({\rm{BH}}\cdot \,\tan \,{\rm{i}})({\rm{n}}/{\rm{c}})\cdot $$

If *θ* = 0°, the TE and TM pulses reach the detector simultaneously, and no delay is observed (Fig. 3c). There is a systematic compensation as already described. When the detector is rotated counter-clockwise (*θ* > 0°), the TE pulse reaches the detector before the TM pulse, as in Fig. 1b. The measured delay between the two pulses is:

3 $$q{\delta }_{Meas}=H^{\prime} B^{\prime} /c=(H^{\prime} A^{\prime} \cdot \,\tan \,\theta )/{\rm{c}}=({\rm{BH}}\cdot \,\tan \,\theta )/{\rm{c}}\cdot $$

From equations (2) and (3) we deduce the actual value of the Newton-Wigner delay time:

4 $$q{\delta }_{NW}=q\delta {\tau }_{Meas}\cdot ({\rm{n}}\,\tan \,i)/\tan \,\theta \cdot $$

Thus, when the detector is rotated so that the finish line A’B’ is more parallel to AB, as for the positive values of *θ* =  + 9° and *θ* =  + 25°, the TE pulse clearly arrives before the TM pulse as expected (Fig. 3c) and Eq. (4) leads to the Newton-Wigner time delay along the interface of *qδτ*_*NW*_ = 2595 ± 30 fs for *θ* =  + 9° for example.

The divergence of the measured time delay values versus the orientation of the finish line is an unavoidable consequence of the spatial shift between the TE and TM pulses, with an inversion point at *θ* = 0°. If we introduce a similar factor of merit for the measured time delay, i.e. *Q*_*NW*_ = *q δτ*_*NW*_/*Δt*_*c*_, where *Δt*_*c*_ corresponds to the time duration of the correlation signal at half-maximum, we obtain the results shown in Fig. 3d. The Rayleigh-like criterion (i.e. *q δτ*_*NW*_ = *Δt*) is satisfied only for $\theta \,\ge 8^\circ$ and the sharp decrease of the *Q* factor around *θ* = 0° excludes any direct measurement in the central zone with the correlation technique. However, high variability of the measured time delays shows the crucial role played by defining the finish line for correctly reaching the true Newton-Wigner time delays.

**Figure 4:**
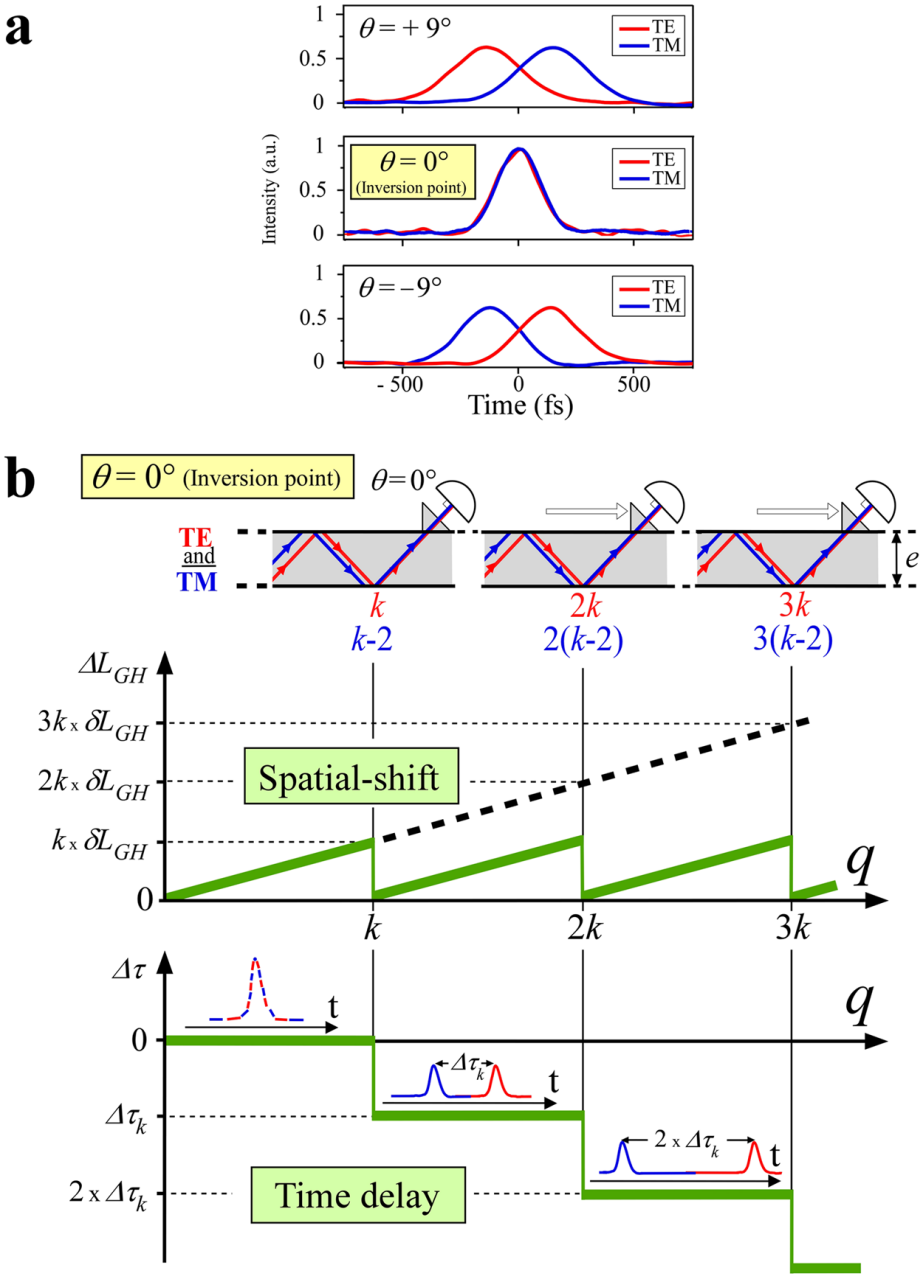
Variability of the measured delay time. **(a)** Change of sign of the measured delay time between the TE and TM laser pulses around the inversion point, successively observed on the intensity correlation profiles for + 9°, 0° and −9° rotations of the detection surface of the swivelling two-photon detector. (**b**) After *q* reflections, for a long waveguide, the TM component catches up with the TE component leading to a cyclical regime for *q* = *k*, 2*k*, 3*k*. If the laser pulse polarization is launched at 45° from the incidence plane of the prism, for *q* varying from 1 to *k*, with the detector oriented at *θ* = 0°, the Newton-Wigner delay between TM and TE is automatically compensated (see text). By contrast, for *q* = *k*, as the spatial shift *ΔL*_*GH*_ is cancelled, the detector receives two pulses separated by a *Δτ*_*k*_ delay including the Newton-Wigner delay (see Supplementary Information). For *q* = 2*k* and 3*k* the delay *Δτ* at *θ* = 0° is multiplied by 2 and 3 respectively.

For negative rotations *θ* of the swivelling detector, the measured values of *qδτ*_*Meas*_ are reversed as shown for *θ* = −9° in Fig. 4a. The TM signal arrives before the TE signal. *θ* = 0° is really an inversion point in our case. In any experiment with a Goos-Hänchen spatial shift along an interface, the large variation of the measured time delays around the inversion point is a necessary signature of the true Newton-Wigner delay. Although the Newton-Wigner delays are essentially positive due to the causality principle, the measured delays can be positive, zero or negative.

### The cyclical regime for the Goos-Hänchen spatial shifts and the Newton-Wigner delays

As suggested by the photograph of Fig. 1a where the TM pulse is already shifted by 0.8 mm compared to the TE pulse after 161 reflections corresponding to a propagation length of about 0.2 m, one may wonder what regime should be expected in longer waveguides (1 m long or more). It turns out that the spatial shifts do not keep on increasing monotonically but exhibit a cyclic behavior. As shown in Fig. 4b after *q* = *k* reflections of the TE pulse, the TM pulse catches up with the TE pulse. However, the TM pulse has experienced only *k*−2 reflections. For an even longer guide such spatial coincidence of the TE and TM pulses is repeated after 2*k* and 2(*k*-2), 3*k* and 3(*k*-2) reflections for the TE and TM pulses respectively. We are left with a cyclical regime for the Goos-Hänchen spatial shift as shown in Fig. 4b. For a given guide and a particular wavelength, one can introduce a Goos-Hänchen beat length (see Supplementary Information Fig. S1) as the distance over which the TM mode will experience an accumulated extra shift compared to the TE mode, such that the beams are again superposed. In this case, at the end of each beat length (for *k*, 2*k*, 3*k*, …), where the spatial shift between the TE and TM pulses is cancelled, a swivelling detector is no longer necessary, and measurements of the time delay can be performed at *θ* = 0°. Moving the extracting prism along the guide, one can record a step pattern schematized at the bottom of Fig. 4b for the time delay that corresponds to the successive beat lengths. The expected measured delays are now systematically negative as the TE pulses undergo an extra-delay corresponding to the time spent for undergoing two more reflections in the guide. The time delay increment *Δτ*_*k*_ between the TE and TM pulses is equal to this TE penalty, minus the true Newton-Wigner delay *kδτ*_*NW*_ accumulated after *k* reflections (see Supplementary Information). When the 2*k* and 3*k* reflections are reached, the guide can work as a Vernier calliper, leading to a potentially higher precision on the time delay for a single reflection at each step.

### The compensation of pulse distortions by the Newton-Wigner delays

**Figure 5:**
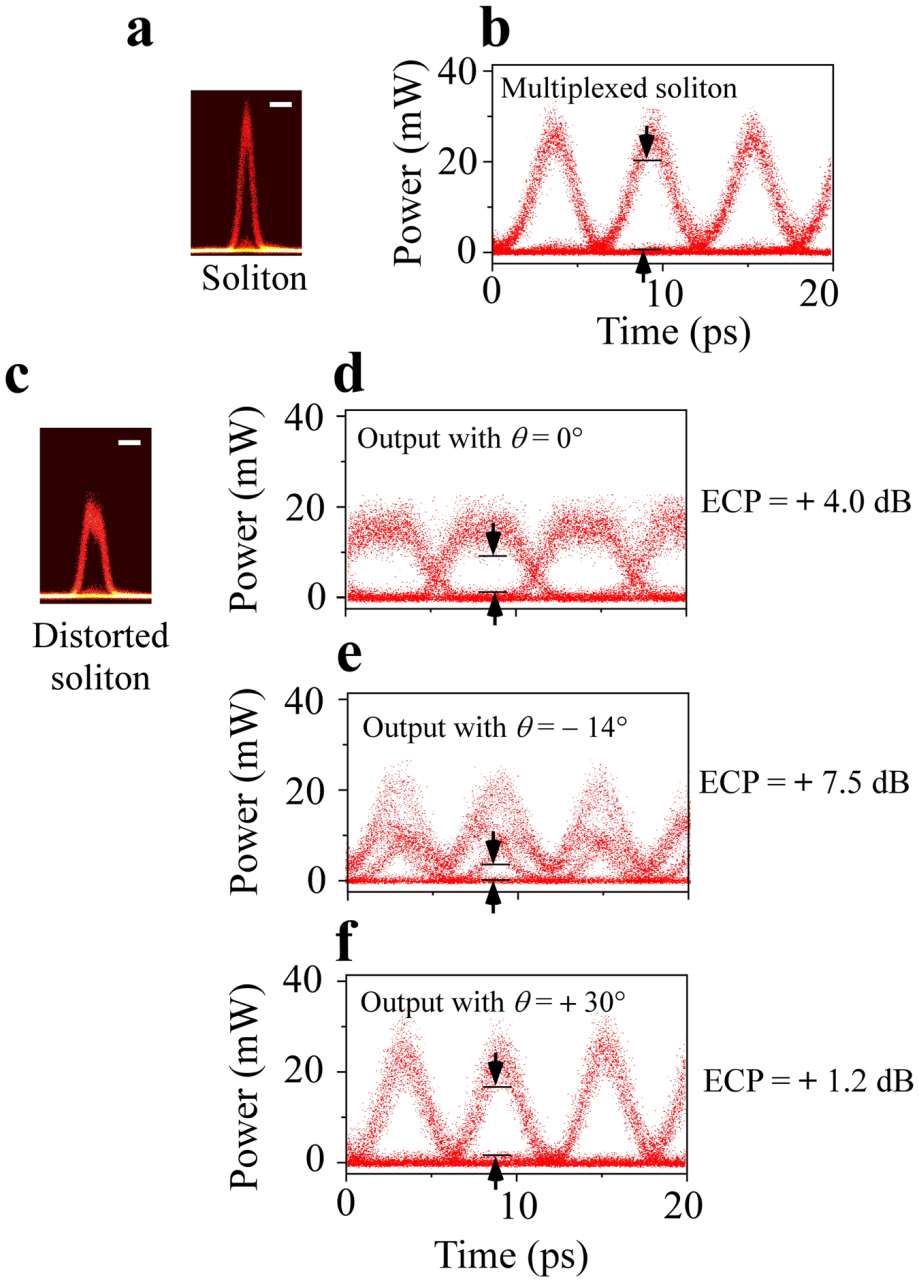
Rectification of a distorted soliton. Eye diagrams for a 160 Gbit/s telecommunication signal at a wavelength of 1550 nm, measured with the forked fibre detector and an optical sampling oscilloscope. **(a)** Picture of a TE polarized soliton entering the waveguide (scale bar, 5 × 10^−12 ^s). **(b)** Multiplexed soliton. **(c)** Picture of a distorted soliton. (**d**), **(e)**, **(f)** Multiplexed signals corresponding to the distorted soliton at the output of the waveguide for different orientations of the finish line of the forked detector: **(d)** *θ* = 0°, **(e)** ***θ*** = −14°, the eye closure penalty is increased, **(f**) *θ* =  + 30°, the eye closure penalty is almost cancelled.

The dynamics of the Newton-Wigner delays offers the possibility of compensating for distortions in the propagation of high-bit-rate signals such as solitons^30^ in optical telecommunication systems with total reflections. Figure 1c schematizes the launching of a soliton in a passive optical guide inserted in the system of Fig. 2. The detector has been modified in order to separately detect the TE and TM contributions of the distorted soliton at 1.55 μm. We used the forked detector which consists of two cleaved single mode fibres, as in a Hanbury-Brown and Twiss like experiment^31^. The two input faces of the fibres define a finish line, as when we dealt with femtosecond pulses. When a typical unperturbed soliton such as the one in Fig. 5a is first multiplexed to obtain the 160 Gbit/s high-bit-rate signals, a large vertical eye opening is recorded only limited by the signal-to-noise ratio (Fig. 5b). For a randomly polarized distorted soliton (at the input) as depicted in Fig. 5c, the eye diagram for the corresponding multiplexed soliton rapidly degrades. The eye diagram at the output is really greatly reduced for *θ* = 0°, showing an eye closure penalty of 4.0 dB (Fig. 5d). For *θ* = −14°, the eye opening is still more reduced leading to a 7.5 dB penalty (Fig. 5e). By contrast, for *θ* =  + 30° the eye diagram is restored, showing a residual penalty of 1.2 dB (Fig. 5f). So, combining the spatial shifts and the associated dynamics of the temporal delays at interfaces optimizes the quality of the detection of high-bit-rate signals. Here the spatial shift between the TE and TM beams reaches 1 mm. In a miniaturized system, the necessary shift could be reduced to 100 μm corresponding to the distance between two joined fibres of the fork. Only 3 cm long guide would then be needed.

### The Newton-Wigner time delay measurements for different particles on interfaces

**Figure 6:**
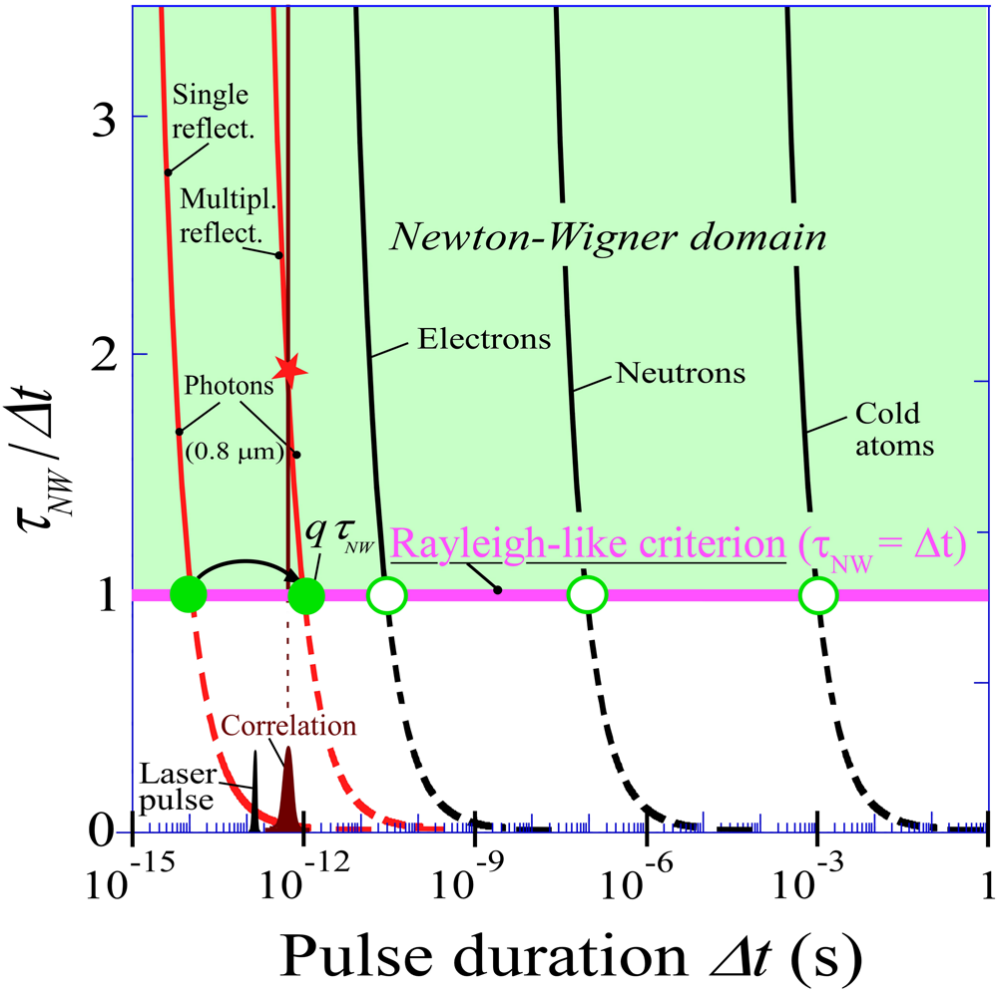
Comparative conditions for Newton-Wigner time delay measurements for light and different particles on interfaces. A direct measurement requires satisfying the Rayleigh-like criterion (pink line), i.e. *Δt* < *τ*_*NW*_, where *Δt* corresponds to the duration of the clock signal to be used for a given expected time delay *τ*_*NW*_. The corresponding working point has to be located in the so-called Newton-Wigner domain. In optics (red curves) using 150 fs laser pulses with a 300 fs correlation signals, the Rayleigh condition is not fulfilled for a single reflection, but only for multiple reflections (the filled green circles represent the experimental values for one and 161 reflections). For particles (black curves), for which correlations are no more necessary, longer pulse durations are sufficient to reach the expected delays (open green circles).

Spatial shifts and time delays observed for transverse waves are also expected for longitudinal and de Broglie waves. Although the spatial shifts and the time delays at interfaces have been predicted for many particles^9, 32–38^, to the best of our knowledge, only the spatial shift for neutrons has yet been observed^19^. The respective dynamics of both quantities observed in optics can bring new insights in the investigations for particles and their de Broglie matter waves. Figure 6 shows the comparative issues to be resolved for measuring the time delay for the optical waves and particles. In optics, while the tiny time delay for a simple reflection is only of the order of 20 fs requiring correlation techniques, the amplification via multiple reflections allows the Rayleigh-like criterion to be fulfilled, so as to reach the Newton-Wigner domain where the measurements are straightforward. As the expected time delays for electrons^32–34^, neutrons^35^ and cold atoms^36, 37^ are considerably larger than those for photons, by about three, seven and eleven orders of magnitude respectively, measuring the delays rather than the corresponding spatial shifts seems more accessible. The Rayleigh-like criterion in Fig. 6 shows that it is much easier for particles to reach the Newton-Wigner domain, i.e. the region where the pulse width Δ*τ* to be used, is shorter than the delay to be measured. While the expected or measured spatial shifts for particles remain tiny, the expected time delays are rather large. Indeed, the speeds of the particles can be considerably reduced, reaching the mm/s range for very cold atoms. For instance metastable Ne and He atoms^36, 37^ and Bose-Einstein condensates^38^ exhibiting quantum reflections at grazing incidence on solid surfaces, seem to be a promising case for isolating their millisecond Newton-Wigner time delays. The triplet state of the He atom^39, 40^ seems to be the best candidate to observe, for the first time, the delay in quantum reflection. Paradoxically, although the expected Newton-Wigner time delays for particles are much larger than those measured in optics and do not require any correlation techniques, they remain to be observed.

## Conclusions

The swivelling detection is a powerful method to unambiguously identify and measure the fundamental Newton-Wigner time delays at interfaces. Combining the respective dynamics of the measured time delays and the accompanying spatial shifts permits us to compensate for distortions in high-bit-rate transmissions. The introduction of the Goos-Hänchen beat length in a long planar waveguide leads to a cyclical regime that opens new possibilities for straightforward measurements of the Newton-Wigner delays. Moreover, in spite of the higher complexity of the particle experimental set-ups, the swivelling detection should enable the Newton-Wigner time delays to be clearly identified for the first time in the Newton-Wigner domain.

## Electronic supplementary material

Supplementary Information
