# Supplementary material for: Dynamics and detection of the Newton-Wigner time delays at interfaces using a swivelling method
Source: Sci Rep. 2017 Aug 22;7:9083. doi: 10.1038/s41598-017-09502-9 (PMC5567374; doi:10.1038/s41598-017-09502-9)
Supplement: Supplementary file 1 — Supplementary Information [file 41598_2017_9502_MOESM1_ESM.pdf]

SUPPLEMENTARY INFORMATION

Dynamics and detection of the Newton-Wigner time delays at interfaces using a swivelling method

Albert Le Floch[1,2,3], Olivier Emile[1,2], Guy Ropars[1,2] & Govind P. Agrawal[4]

[1]Laboratoire de Physique des Lasers, UFR SPM, Université de Rennes 1, 35042 Rennes, France.

[2]Université Bretagne Loire, 35044 Rennes cedex

[3]Laboratoire d'Electronique Quantique et Chiralités, 20 square Marcel Bouget, 35700 Rennes, France.

[4]The Institute of Optics, University of Rochester, Rochester, New York 14627, USA.

Correspondence to guy.ropars@univ-rennes1.fr

I- The Newton-Wigner delays in long waveguides

Let us consider the waveguide schematized in Figure S1 with a laser beam linearly polarized at 45° from the incidence plane of the input prism. When launched at an incidence angle i near i_C, corresponding to the guide critical angle defined by $\sin i_C = 1/n$, the TE and TM components experience two spatially separated paths as shown in Figure S1. For a given wavelength λ and a given thickness e of the guide, the TM mode catches up with the TE mode after q reflections for TE but only after q-2 reflections for TM (here $q = k = 9$). At point B, the TE and TM beams are again superposed as in A, defining the Goos-Hänchen beat length. For a longer waveguide the beam superposition is also produced for $q = 2k$, $3k$… leading to the so-called cyclical regime.

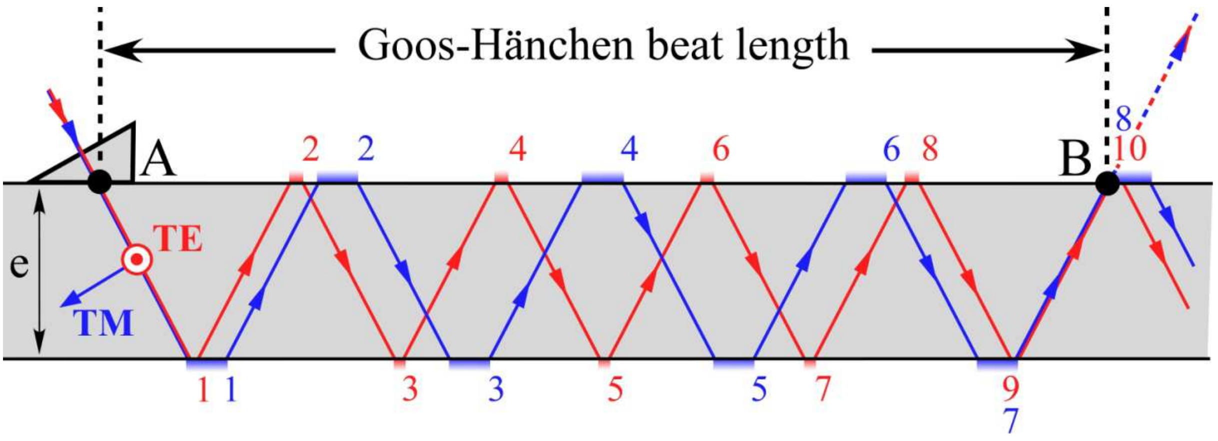

Figure S1 Scheme defining the Goos-Hänchen beat length. After q reflections of the TE pulse, but only q-2 reflections of the TM pulse, the initial spatial superposition of the two beams is recovered.

Surprisingly, in the pulsed regime, for $q = k$, $2k$, $3k$ reflections, as the spatial shifts between TE and TM are cancelled, we can directly measure the delays between these TE and TM pulses. The correlation using the two-photon detector will exhibit separated TE and TM signals at point B, even for $\theta = 0°$ as shown in Figure 4b. However, in the cyclical regime, the measured $\Delta\tau_k$ delay between TE and TM is always negative and equal, for $i \cong i_C$, to $\Delta\tau_k = -\frac{2\,e}{c.\cos i_C}\left(+n - \frac{1}{n}\right)$, where c is the speed of light in vacuum. The first term represents

the extra delay encountered by the TE pulse for its two supplementary reflections, while the second term represents the cumulated Newton-Wigner time delay after $q = k$ reflections. For a long waveguide corresponding to several Goos-Hänchen beat lengths, the theoretical curves in Figure 4b for the total delay between TE and TM pulses show the successive gaps for $q = k$, $2k$, $3k$. It is worth noting that although the time spent by the TM mode outside the waveguide is larger than the time spent by the TE mode, the TM mode reflections are detected before the TE mode reflections.

II- The Newton-Wigner delays in polarization-maintaining fibres

Polarization-maintaining optical fibres are used in special applications where preserving polarization is crucial[1]. During the propagation along the axis, the waves undergo several total reflections. These fibres are anisotropic single-mode optical fibres, with two orthogonal polarization modes. When a linearly polarized light is launched into the fibre along the slow axis for instance, as shown in Fig. S2, the wave behaves as a TM mode for the points A and A' along the slow axis, but as a TE mode for the points B and B' along the fast axis. The different Newton-Wigner delays along the two axes induce deteriorations of the propagating wave, which could be compensated by periodically alternating the fiber axis orientations. Note that at points like C, small ellipticities can be introduced in the wave polarization along this system.

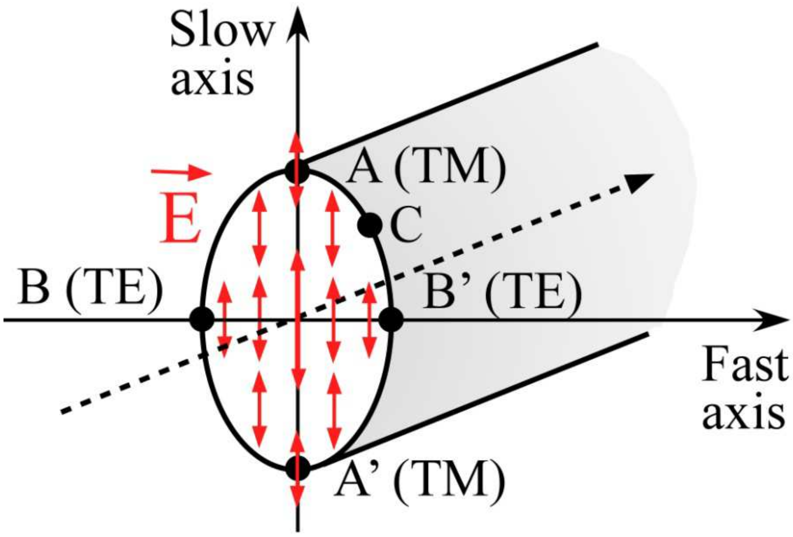

Figure S2 Scheme of a linearly polarized beam injected along the slow axis of a polarization-maintaining fibre.

III- The delays in time-resolved experiments

Today, fast time-resolved experiments dealing with evanescence occurs are of great interest in many areas where wave and particle pulses interact. In photon-induced near-field electron microscopy, where the evanescent fields are imaged by electron pulses[2], the temporal overlap of laser and electron pulses requires robust methods taking account of the different delays[3]. The high temporal resolution can only be limited by plasmon lifetimes and nonlinearities[4]. Nonlinearities of the Newton-Wigner delays have also been observed at interfaces[5]. Moreover in attosecond metrology, time delays in photoemission are able to discriminate small differences in the timing of electron emission from different quantum states[6,7]. Real-time insights into fast microscopic phenomena in atoms or molecules have also been achieved[8]. Besides, the asymmetry of such time delays in the ionization of chiral molecules has even been predicted[9].

REFERENCES

1. Agrawal, G. P. "*Nonlinear Fiber Optics*" (5th ed. Academic Press, Oxford, 2013).

2. Barwick, B., Flannigan, D. J. & Zewail, A. H. Photon-induced near-field electron microscopy. *Nature* **462**, 902-906 (2009).

3. Scoby, C. M., Li, R. K. & Musumeci, P. Effect of an ultrafast laser induced plasma on a relativistic electron beam to determine temporal overlap in pump-probe experiments. *Ultramicroscopy* **127**, 14-18 (2013).

4. Plemmons, D. A., Park, S. T., Zewail, A. H. & Flannigan, D. J. Characterization of fast photoelectron packets in weak and strong laser fields in ultrafast electron microscopy. *Ultramicroscopy* **146**, 97-102 (2014).

5. Loas, G., Bonnet, C., Dunseath, K., Chauvat, D., Emile, O. & Le Floch, A. Magic angle detection of nonlinear Newton-Wigner times at interfaces. *EPL* **77**, 64003 (2007).
6. Schultze, M. et al. Delay in photoemission. *Science* **328**, 1658-1662 (2010).
7. Krausz, F. & Stockman, M. I. Attosecond metrology: from electron capture to future signal processing. *Nat. Photon.* **8**, 205-213 (2014).
8. Bourgain, R., Pellegrino, J., Jennewein, S., Sortais, Y. R. P. & Browaeys, A. Direct measurement of the Wigner time-delay for the scattering of light by a single atom. *Opt. Lett* **38**, 1963-1965 (2013).
9. Chacon, A., Lein, M. & Ruiz, C. Asymmetry of Wigner's time delay in a small molecule. *Phys. Rev. A* **89**, 053427 (2014).
